# The Current Cardio-Oncology Landscape Across Australia

**DOI:** 10.1007/s11864-026-01390-4

**Published:** 2026-04-18

**Authors:** Daniel H. Chen, Lloyd Butel-Simoes, Mitchum Bower, Mark T. Nolan, Sanjeev S. Kumar, Trent D. Williams, Bogda Koczwara, Philippa Ell, Joshua D. Bennetts, Gayathri R. Nair, John Lee, Satish Ramkumar, Doan T. M. Ngo, Aaron L. Sverdlov

**Affiliations:** 1 Prince of Wales Hospital, Randwick, Sydney, NSW 2031 Australia; 2 Faculty of Medicine and Health, University of New South Wales, Sydney, NSW 2052 Australia; 3 St George Hospital, Kogarah Sydney, NSW 2217 Australia; 4 Cardiovascular Department, John Hunter Hospital, Newcastle, NSW 2305 Australia; 5 College of Health, Medicine and Wellbeing, The University of Newcastle, Callaghan, NSW 2308 Australia; 6 Newcastle Centre of Excellence in Cardio-Oncology, Newcastle, NSW 2305 Australia; 7 Hunter Medical Research Institute, New Lambton Heights, NSW 2305 Australia; 8 Peter MacCallum Cancer Centre, Melbourne, VIC 3000 Australia; 9 Baker Heart and Diabetes Institute, The University of Melbourne, Parkville, VIC 3010 Australia; 10 Chris O’Brien Lifehouse, Camperdown, Sydney, NSW 2050 Australia; 11 Royal Prince Alfred Hospital, Camperdown, Sydney, NSW 2050 Australia; 12 Garvan Medical Institute of Medical Research, Sydney, Australia; 13 School of Nursing and Midwifery, College of Health, Medicine and Wellbeing, University of Newcastle, Callaghan, NSW 2308 Australia; 14 Australian Research Centre for Cancer Survivorship, Sydney, Australia; 15 GenesisCare, Newcastle, Gateshead, NSW 2290 Australia; 16 School of Biomedical Science and Pharmacy, University of Newcastle, Newcastle, Australia; 17 Victorian Heart Hospital, Monash Victorian Heart Institute, Monash University, Clayton, Melbourne, 3168 Australia

**Keywords:** Cardio-oncology, Australia, Epidemiology, Models of care, Service delivery

## Abstract

Cardio-oncology is a rapidly evolving subspecialty at the intersection of cancer and cardiovascular disease (CVD), two of Australia’s leading causes of morbidity and mortality. Advances in cancer detection and therapy have markedly improved survival, resulting in a growing cohort of more than 1.2 million Australian cancer survivors. However, these patients are at increased risk of cardiovascular toxicity from cancer therapies, with CVD now surpassing recurrent malignancy as a leading cause of late mortality. In response, several quaternary and tertiary Australian public hospitals have established cardio-oncology services through grassroots initiatives, this has evolved into structured clinics embedded within tertiary hospitals. Despite increasing recognition and demand, access remains uneven, with services concentrated in metropolitan centres and limited provision for rural, remote, and Indigenous populations. National professional societies, including the Cardiac Society of Australia and New Zealand (CSANZ) and the Clinical Oncology Society of Australia (COSA), have recently established collaborative working groups to advance education, guidelines, and advocacy. Australia has also made significant research contributions through registries such as the Australian Cardio-Oncology Registry (ACOR) and clinical trials including SUCCOUR, BREXIT, and SMART-BREAST. Looking forward, key priorities for the Australian cardio-oncology community have surfaced including scaling workforce capacity, integrating structured education and training, expanding outreach and telehealth models, and addressing health inequities in underserved populations. These priorities along with upcoming initiatives such as the ACORES symposium will play an essential role in developing this sub speciality field. This review provides a comprehensive overview of the current cardio-oncology landscape across Australia, outlining its development, achievements, challenges, and future directions.

## Introduction

Cardiovascular disease (CVD) and cancer represent the two most common causes of mortality both globally, and in Australia [1]. Cancer is an independent predictor of cardiovascular morbidity and mortality, in part due to the cardiotoxic effects of many modern oncological therapies, but also because the two pathologies share a number of common risk factors [2]. Advances in cancer detection and treatment have resulted in an increase in both the numbers of cancer survivors as well as the duration of cancer survivorship. The discipline of cardio-oncology was borne out of the growing need to address cardiovascular morbidity and mortality in cancer. This has led to grassroots efforts of committed clinicians in Australia who established early services that have gradually expanded into structured, multidisciplinary clinics embedded within tertiary hospitals. The subsequent growth of such services has been driven by increasing demand, reflecting both the expanding cancer survivor population and the recognition of cardiovascular toxicity as a critical determinant of long-term outcomes [3]. In additional to its growth within the clinical space, Australia has contributed significantly to the global cardio-oncology research agenda, with landmark studies such as SUCCOUR, BREXIT, and SMART-BREAST, as well as the establishment of ACOR, a multicentre registry capturing real-world practice and outcomes [3–7].

Despite this progress, several challenges persist including access disparity experienced by Indigenous and rural populations, and insufficient workforce capacity [8–10]. National bodies such as the Cardiac Society of Australia and New Zealand (CSANZ) and the Clinical Oncology Society of Australia (COSA) have partnered to establish joint working groups aimed at developing guidelines, advancing education, and advocating for service expansion. Leading clinicians and researchers have more recently also collaborated as part of the Australian Cardio-Oncology Research and Education Society (ACORES) to further that agenda [11]. 

This review provides a comprehensive overview of the current cardio-oncology landscape across Australia. We describe the epidemiology of cancer and cardiovascular disease, the historical development of services, current models of care, training and workforce considerations, research contributions, and the barriers that continue to limit equitable access. Finally, we outline future directions for the discipline, highlighting opportunities to expand services, strengthen multidisciplinary collaboration, and ensure equitable access for all Australians living with cancer and cardiovascular disease.

## Section 1: Epidemiology of Cardio-Oncology in Australia

In Australia, the leading causes of mortality are cancer and cardiovascular disease (CVD), accounting for approximately 30% and 24% of deaths, respectively [12, 13]. Cancer and cardiovascular disease co-exist in a complex relationship, each increasing the risk of the other, and with many shared risk factors [14]. In keeping with Australia’s ageing and expanding population, the number of new cancer diagnoses increased by 88% between 2000 and 2023. Despite this, cancer survivorship has also increased due to early detection and improved treatment outcomes [12]. 

The increasing numbers of cancer diagnoses, complexity of cancer treatments, novel cancer treatments, and number of lines of cancer treatments administered has subsequently been accompanied by increasing cancer treatment related cardiovascular toxicity (CTR-CVT), with its incidence estimated between 10 and 20% [15]. Concurrently, improvements in cancer screening and diagnosis and advances in the contemporary cancer treatment landscape have seen the rates of cancer survivorship increase. More than 70% of those diagnosed with cancer in Australia are now survive five years following their diagnosis, resulting in more than 1.2 million Australian cancer survivors [12]. This increasing number of cancer survivors further represents an at-risk cohort who are more than 40% more likely to develop cardiovascular disease than their age matched counterparts [16]. Whilst recurrent cancer had previously been the main cause of mortality in cancer survivors, the South Australian Cancer Registry data has demonstrated that 55% of deaths in cancer survivorship are attributable to non-cancer related causes [17]. Ischaemic heart disease is the leading cause of non-cancer related mortality, with cardiovascular mortality exceeding that of cancer mortality by 13 years following a diagnosis of cancer [17]. Cancer survivors therefore warrant targeted and subspecialist multidisciplinary care to reduce cardiovascular morbidity and mortality during cancer survivorship.

## Section 2: History and Development of Cardio-Oncology in Australia

Cardio-oncology in Australia originated from multiple grass-roots initiatives from passionate individuals that slowly coalesced into first local, then over several decades into statewide, and more recently, national movements aimed at improving cardiovascular care for cancer patients. One of the earliest calls to established dedicated cardio-oncology services was published nearly 40 years ago [18]. However, the formal and systematic delivery of cardio-oncology care in Australia was long hindered by the absence of societal guidelines, challenges with funding clinics and often a lack of awareness amongst oncology and cardiology professionals regarding this subdiscipline.

Over the past decade, cardio-oncology in Australia has matured from a loose collection of interested clinicians into structured hospital-based services. National-level data is scarce, but several centres have published their experience in establishing new services with the emergence of common themes. Local data suggests that up to half of all patients who develop a cardiomyopathy after chemotherapy are not referred to a cardiology service. Qualitative research has demonstrated a lack of patient awareness of possible cardiac side-effects of anti-cancer therapy, and a strong appreciation for expert cardio-oncology care [19].

**Fig. 1:**
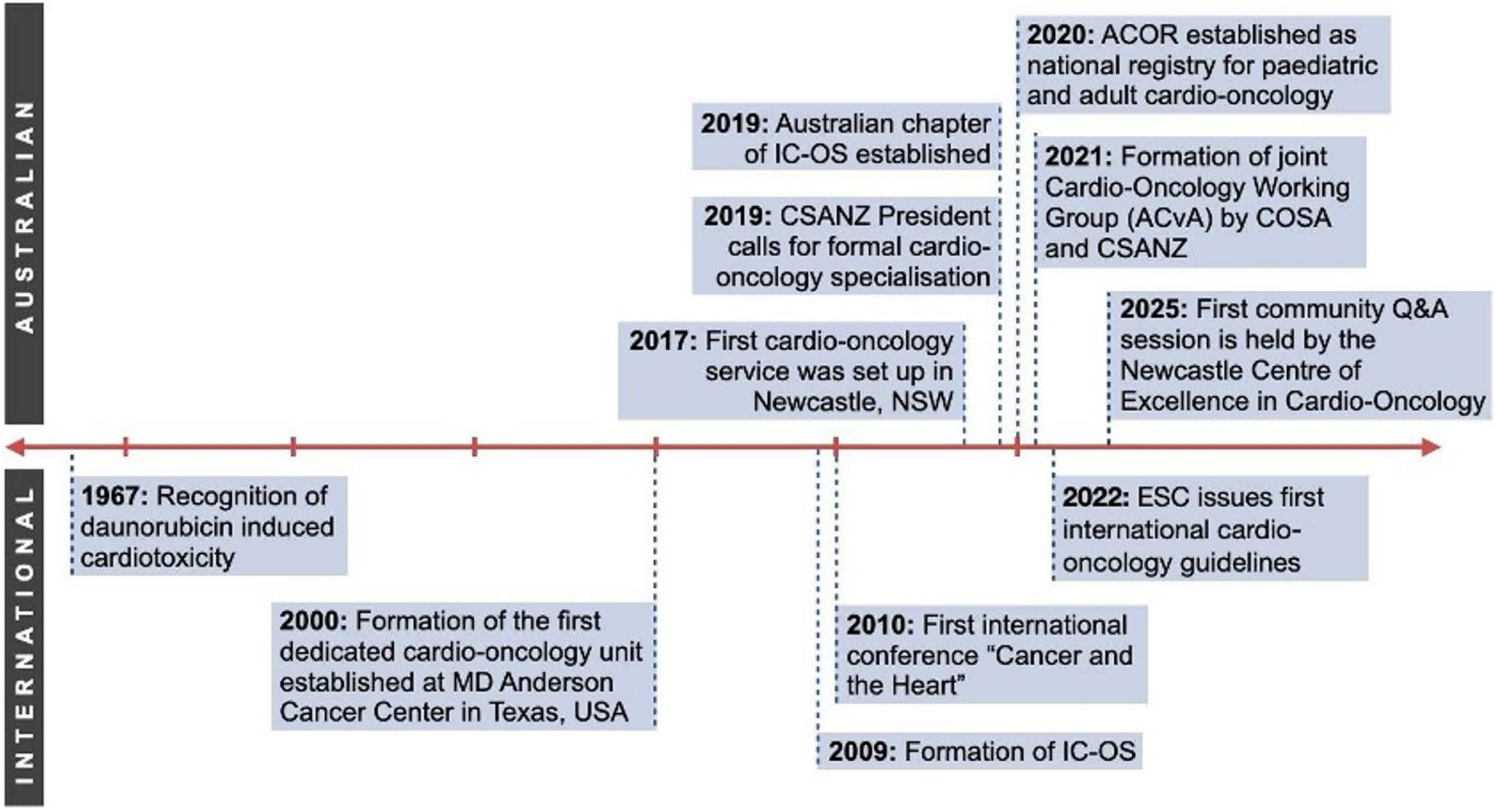
Timeline of the development of the Australian and international cardio-oncology landscape

Key international and Australian milestones in the development of the field are summarised in Fig. 1. The first Cardio-Oncology service was set up in Newcastle in New South Wales in 2017 and has since served as a template for other centres. This organic, clinician drive growth mirrors a similar pattern seen in the United States of America (USA) [20]. Many centres reported unexpectedly high demands following service initiation, often presenting capacity and resourcing challenges [21]. A survey of hospital cardiology department directors in 2021 revealed that although 70% of departments lacked formal cardio-oncology services, 48% attributed this to scarce funding and 70% had a plan to commence such services in the near future [22]. Provision of cardio-oncology services at hospitals has continued to grow and has now expanded to the paediatric setting [23].

In the last five years, there has been growing momentum towards a co-ordinated national cardio-oncology framework. In 2021 the Cardiac Society of Australia and New Zealand (CSANZ) and Clinical Oncology Society of Australia (COSA) set up a working group to formalise collaboration in managing cardiovascular disease in the cancer population [11]. Long-term survivorship clinics have also proliferated, providing the longitudinal care that earlier cardio-oncology services lacked. Strategic leadership in cardio-oncology research is now evident at the national level, supported by multiple professional bodies including the Australian Cardiovascular Alliance, a non-profit consortium spanning the translational spectrum from drug discovery to implementation science [3]. 

## Section 3: Distribution and Referral Catchments for Cardio-Oncology Clinics in Australia

Despite increasing demand, dedicated cardio-oncology services remain concentrated in major metropolitan centres, often embedded within tertiary hospitals that may also provide inpatient consultation [24]. New South Wales has the greatest number of cardio-oncology programs. There are currently four sites in Australia recognized by the International Cardio-oncology Society (ICOS) as a “Gold” Centre of Excellence:

- Newcastle Centre of Excellence in Cardio-Oncology, covering 4 institutions: Calvary Mater Newcastle, Hunter New England Health, Hunter Medical Research Institute and the University of Newcastle and the private clinic at Newcastle Heart (Newcastle, NSW),
- Victorian Heart Hospital (Melbourne, VIC),
- Prince of Wales Hospital (Sydney, NSW).
- St George Hospital (Sydney, NSW) [24].

One site holds “Silver” Centre of Excellence accreditation – Central Sydney Cardiology (Sydney, NSW). Additional cardio-oncology services operate at:

- New South Wales: Liverpool Hospital (NSW), Nepean Hospital (NSW).
- Victoria: Victorian Comprehensive Cancer Centre, Austin Hospital, St. Vincent’s Hospital, Advara Private, Australian Cardiovascular Specialists.
- Queensland: St. Vincent’s Private Hospital.
- South Australia: Advara HeartCare.

**Fig. 2:**
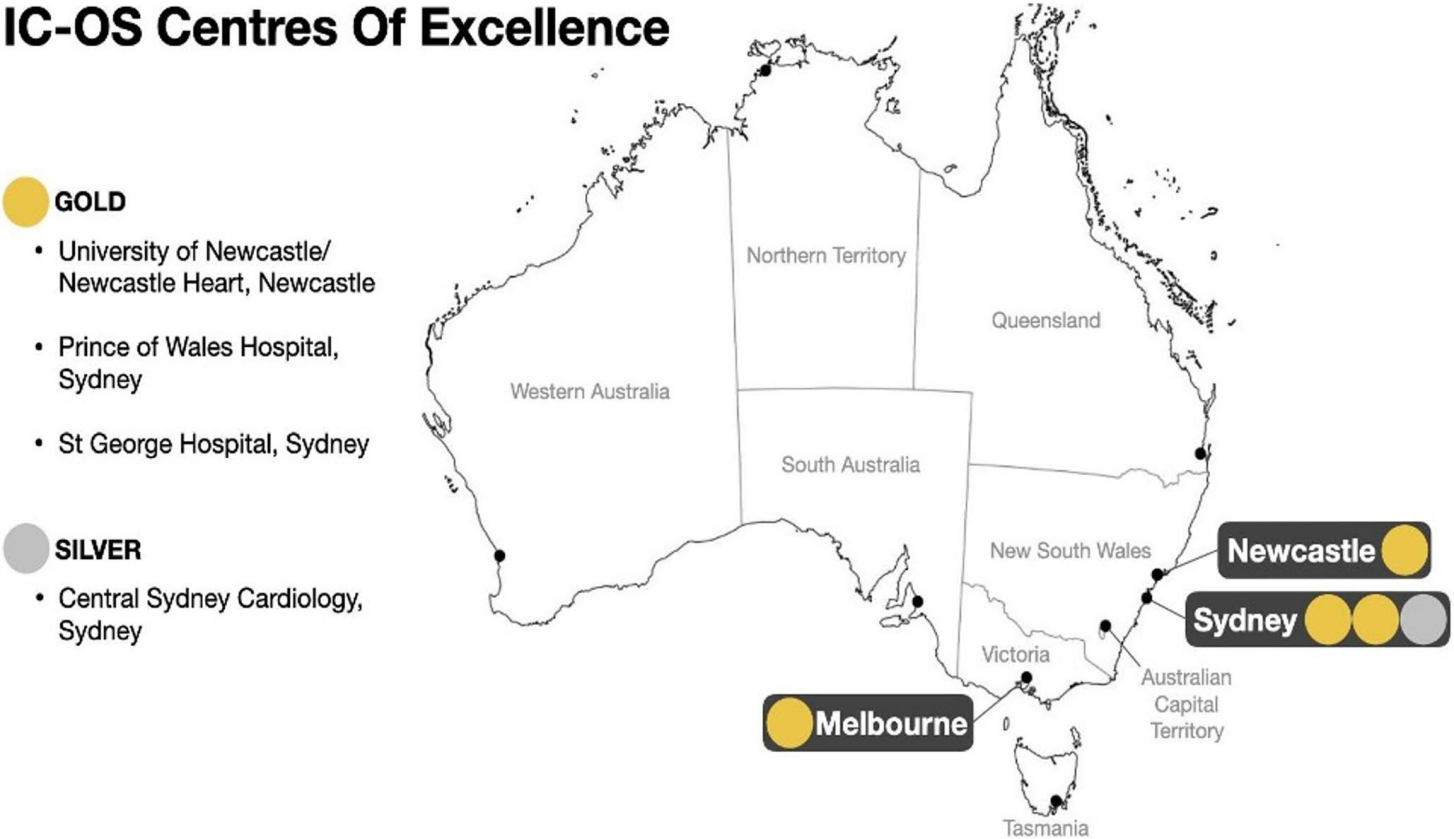
Australian cardio-oncology landscape map based on IC-OS certification

The geographic distribution of ICOS-accredited Centres of Excellence is illustrated in Fig. 2. Beyond Newcastle, Sydney and Melbourne, there are currently no accredited cardio-oncology services within public hospitals. Most patients in regional and remote areas are managed by general cardiology or heart failure services, with complex cases referred to ICOS-accredited tertiary centres. This creates a hub-and-spoke model of care, in where metropolitan specialist hubs support peripheral areas through structured referrals and shared-care pathways. This model reflects the current Australian context, where service availability is shaped by both geographic and funding limitations [11, 22, 25].

Referrals to cardio-oncology clinics are mostly initiated by medical oncologists, haematologists and radio-oncologists, particularly for patients receiving high-risk therapies such as anthracyclines, HER-2 targeted therapies, fluoropyrimidines, tyrosine kinase inhibitors, immune checkpoint inhibitors, or in patients undergoing stem cell transplantation [21]. Paediatric services show a similar trend [23]. Referrals from general practitioners comprise < 10% but expected to rise along with the increase awareness of the importance preventative cardiovascular care in cancer survivors [25]. 

Innovative outreach models to bridge access gaps are emerging, such as the Hunter New England Health outreach initiative which has demonstrated the feasibility and impact of rural outreach and nurse-led care through telehealth consultations and regional clinics [11]. Broader implementation of similar initiatives in other states may help address persistent geographic inequities. Sustainable expansion of cardio-oncology services will require investment in workforce capacity, clearer clinical pathways, and integration across cardiology, oncology, and primary care. Joint efforts by national societies such as the Clinical Oncology Society of Australia (COSA) and CSANZ are already advancing this agenda through education, advocacy, and collaborative planning [11]. 

## Section 4: Cardio-Oncology Education and Training in Australia

There remain significant challenges for education and training; most oncology and cardiology trainees receive limited exposure to cardio-oncology during formal training and practicing clinicians report learning needs in this area. Only 11% of Australian cardiology training programs include structured education and training in cardio-oncology, highlighting limited exposure during training [3]. This is a global issue as half of all medical institutions included in a global survey also lack formal cardio-oncology training for their trainees [26]. Additionally, 88% of cardio-oncology nurses in another global survey have no formal education in cardio-oncology [27]. 

Absent formal education pathways contribute to variability in practice, ultimately leading to fragmented care. The desire for improved education and integrated care is seen from both provider and patient feedback surveys in a qualitative Australian study which found that while patients valued early cardio-oncology input, they perceived shortcomings in ongoing cardiovascular risk education and lifestyle [21]. 

The COSA and CSANZ Cardio-Oncology Working Group sought to improve provider knowledge through conjoint educational meetings and access to educational resources for health professionals [11]. Many Australian cardio-oncology centres rely on informal local initiatives and local health funding to develop their own programs to progress education and training. Trainees often undertake higher degrees by research or seek out major centres for fellowship opportunities across the country or abroad for experience. Newcastle Cardio-Oncology is the only Australian centre offering a dedicated cardio-oncology training fellowship. Other pre-existing cardio-oncology services are considering a similar setup.

Importantly, cardio-oncology modules must be integrated into existing cardiology and oncology training programs to enhance awareness and competence. This may include developing structured cardio-oncology training pathways, involving dedicated cardio-oncology rotations or a set number of mentored clinics as a basic training requirement. The COSA CSANZ Cardio-Oncology Working Group have co-designed e-learning modules to educate health professionals (including on the care of Indigenous populations in trials) [3]. There is also a push for multi-disciplinary education: engaging general practitioners, oncology and cardiac nurses and allied health staff in cardio-oncology training to widen the skilled workforce.

Australia’s cardio-oncology education is progressing via society-led initiatives and exemplary centres across the country; however, education still lacks a unified formal pathway. Bridging this gap through structured training and accreditation is a priority for the coming years, to ensure all cancer patients have access to clinicians with the specialised knowledge to treat cancer whilst providing CVD prevention and both short term and long-term management.

## Section 5: Australian Cardio-Oncology Research Past, Present and Future

Australia contributed substantially to global cardio-oncology through population studies, translational science, and clinical trials. Dedicated research hubs include the Baker Heart and Diabetes Institute, the Victorian Heart Institute and Monash University, (clinical trials and imaging), Murdoch Children’s Research Institute (paediatric cardio-oncology, registry and genetics), and the Newcastle Centre of Excellence in Cardio-Oncology (bench-to-bedside translational science), Prince of Wales and St George Hospitals (clinical research), and the Heart Research Institute (preclinical and biomarker platforms). Together, these programs form the backbone of an expanding national cardio-oncology research network.

### Past and Present Research

Early Australian studies have established the high exposure of our cancer patients to cardiotoxic medications [28], the high burden of cardiovascular disease in cancer survivors unique to our population [17], and significant undertreatment of cardiovascular risk factors [29, 30] prompting call-to-arms initiatives [3, 11]. Current research spans prevention, surveillance, and mitigation of cardiotoxicity.

Recent landmark trials have been highlighted in Table 1. SUCCOUR-MRI and ALLO-Active are two particular trials that have changed clinical management. SUCCOUR-MRI trial demonstrated that initiation of ACEi/ARB plus β-blocker therapy when GLS worsens before LVEF decline reduces MRI-LVEF deterioration in anthracycline-treated patients [5]. ALLO-Active showed that a structured aerobic + resistance exercise program during and after allogenic-SCT preserved cardiorespiratory fitness, undetected by resting echo or biomarkers [33].

**Table 1:** Key Australian cardio-oncology trials

Study (Authors, Year)	Population	Main Findings	Limitations
Koczwara et al., 2021 (Late mortality study) [17]	32,646 cancer survivors in South Australia (diagnosed 1990 s; ≥5-year survivors)	26.7% of all deaths and 56% of non-cancer deaths were due to CVD in long-term survivors. CVD mortality was significantly higher than in age-matched general population (SMR ~ 1.24), becoming the leading cause of death > 13 years post-diagnosis.	Retrospective cohort: older treatment era (1990s data) may not reflect modern therapies. Lacked granular treatment information; single-state registry.
SUCCOUR Trial (Thavendiranathan et al., 2021) [31]	311 adults on anthracycline chemo (international multi-center RCT)	Strain-guided cardio-protection (echo GLS surveillance with early ACEi/β-blocker) vs. usual care showed *no significant difference in LVEF decline* at 1 year. However, patients receiving cardioprotective therapy had significantly less LVEF reduction than controls, suggesting benefit in preventing subclinical decline.	Follow-up 1 year only, focused on LVEF (no long-term clinical endpoints). Potential underpower to detect differences in hard outcomes; intervention uptake varied.
BREXIT Trial (Foulkes et al., 2023) [6]	104 women with early breast cancer on anthracyclines (single-center RCT)	A 12-month supervised exercise program (aerobic + resistance training) was compared to guideline-based physical activity advice. Primary outcome: VO₂ max at 12 months – *no overall difference* between groups. However, exercise adherence correlated with preserved fitness and functional capacity decline was prevented.	Single-site study; moderate sample size. Adherence was suboptimal (only subset got full “dose” of exercise). VO₂ may not capture all benefits; study not blinded.
SMART-BREAST Trial (Murphy et al., 2023) [7]	103 women with early breast cancer (single-center RCT)	Tested a smartphone app-based exercise intervention vs. usual care. **Primary outcome**: 6-minute walk distance at 12 months – the intervention group had a significant improvement (+ 46 m vs. + 8 m in controls, *p* < 0.001)	Single centre and short-term (12-month) outcome. App engagement levels varied. Clinical impact (e.g. on long-term cardiac events) not assessed; specific to a relatively healthy early cancer cohort.
		Demonstrated that a digital health strategy can improve functional capacity during adjuvant therapy.	
Australian Cardio-Oncology Registry (ACOR) (Conyers et al., initiated 2018) [32]	~ 600 patients per year across 13 Australian sites (prospective registry; includes adult cancer patients on potentially cardiotoxic therapy)	Established a national cardio-oncology database linking oncology and cardiology data. Captures detailed treatment exposure and cardiac outcomes, enabling real-world analyses of cardiotoxicity and care delivery. Has facilitated new cardio-oncology clinics and provides data for cost-effectiveness and outcome studies (via linkage to Medicare/PBS).	Not a controlled trial – observational data subject to referral bias (patients in participating centers). Still in early stages: long-term outcomes pending. Coverage not yet truly nationwide (centers mostly in NSW/VIC).
SUCCOUR-MRI (Marwick et al., 2024) [5]	Prospective multicentre randomized control trial across 14 international sites, 355 adult patients undergoing anthracycline-based chemotherapy with atleast one CTRCD risk factor. 105 patients with a reduced GLS (> 12%) but not a reduced LVEF where randomised to cardio-protection (ramipril and metoprolol) vs. standard care. Primary outcome: A change in cardiac magnetic resonance (CMR) LV ejection fraction from baseline to 1 year	After cardio-protection only 1/49 developed 12 month LVEF-CTRCD compared with 6/56 in the usual care group. The cardio-protection group showed significant improvement in GLS at 3 months post randomization versus little change in the usual care.	Trial had limited power to assess this secondary outcome. Follow-up LVEF was used as a surrogate of HF risk. Initial intention to randomize 140 patients however numbers reduced due to the COVID-19 pandemic resulting in 105 patients and reduced statistical power with wider confidence internals for the effect size. This did not effect the final outcomes.
		Patients with isolated reduced GLS post anthracycline therapy and at least one CTRCD risk factor had better preservation of MRI-LVEF at 12 months when treated with cardio-protective therapies.	Expectedly, patient deaths due to cancer and patients unable to have follow up MRI due to morbidity requiring a limited amount of patients to undergo 3D echocardiography in place of MRI (part of study design).
			Despite randomization, more patients with early change of GLS were allocated to cardio-protection arm, and more patients with late changes of GLS were allocated to usual care arm. As patients were followed over a year from the start of imaging surveillance, people developing change late may not have had time to recover.
			Study utilised standard neurohormonal blockade cardio-protection therapies and did not include other potential cardio-protective agents.
			Finally the GLS criteria used in this study (> 12%) differ from the current guideline cut-off of 15%. (study was designed pre 2022).
ALLO-ACTIVE [33]	Sixty-two adults scheduled for allo-SCT were randomized to either a 4-month activity program (*n* = 30) or usual care (UC, *n* = 32). The activity included multicomponent exercise training (3x/week) and sedentary behaviour reduction (≥ 30 min/day), delivered during hospitalization (approximately 4 weeks) and for 12 weeks post-discharge.	Results: Of 62 participants, 52 (84%) completed follow-up (25 activity, 27 UC). Median adherence to the activity program was 74%.	Limitations include single-site recruitment and lack of allocation concealment, which may introduce a potential Hawthorne effect in the UC group. Recruitment bias toward physically active patients may also have diminished the observed effects of the activity program. Additionally, reliance on Fitbit for measuring inactivity and prompting movement could be limited by proprietary programming changes. Finally, the use of research-grade wearables may have underestimated moderate-to-vigorous physical activity (MVPA) and overestimated sedentary time, as they are insensitive to non-ambulatory exercises and were only used post-intervention.
	Physiological assessments included: (1) Cardiopulmonary exercise testing (VO₂ peak) (2) Exercise cardiac MRI (3) Echocardiographic measurement of left ventricular ejection fraction and global longitudinal strain and (4) Cardiac biomarkers (troponin-I and B-type natriuretic peptide).	In the UC group, VO₂ peak declined by − 3.4 mL‧kg⁻¹‧min⁻¹ (95% CI, − 4.9 to − 1.8), while the decline was attenuated in the activity group (− 0.9 mL‧kg⁻¹‧min⁻¹; 95% CI, − 2.5 to 0.8; interaction *P* = 0.029). Activity also preserved exercise cardiac function compared to UC group on CMRI indices. No treatment effects were observed on cardiac biomarkers or echocardiographic indices.	
COP-RCT (ongoing) (ACTRN12621000928819)	Aiming for 70 adults who have recovered from CTRCD. Single site (hospital)		
	Supervised withdrawal of cardioprotective therapies; vs. usual care (continuing cardioprotective therapies)		
	Difference in composite end point of relapse in cardiac dysfunction at 6 months, defined by: (1) reduction in 3D LVEF between 10% and 50%; (2) heart failure requiring hospital admission; or (3) worsening heart failure requiring medical treatment.		
SOCRATES trial (ongoing) (NCT05180942)	Target for 180 adult patients with melanoma treated with immune checkpoint inhibitors across 12 sites (hospitals).		
	40-mg atorvastatin; vs. usual care (no treatment)		
	Progression of atherosclerosis measured by computed tomography coronary angiography CTCA at 4 weeks		
MONITOR HER2 trial (Ongoing) (ACTRN12624000148572p)	Single centre randomised controlled trial of 3 monthly vs. 6 monthly echocardiographic surveillance for HER-2 positive breast cancer patients. Primary outcome - Echocardiographic derived LV ejection fraction at 12 months.		
IMPACT-CVD (Ongoing) (ACTRN12624001005549)	IMPlementation and Prospective evAluation of dedicated Cardio-oncology services for prevention, monitoring and Treatment of CardioVascular Diseases in patients living with, through and beyond CANCER (IMPACT-CVD in CANCER trial).		
	Each site will establish a nurse-led, physician-supported cardio-oncology risk stratification clinic integrated with cancer services. The clinic will deliver guideline-directed baseline risk assessment, preventive management, and structured surveillance during and after cancer treatment. Surveillance frequency and follow-up will be tailored to individual patient risk, in accordance with international cardio-oncology guideline recommendations.		
	Stepped wedge-controlled trial with a hybrid II design. The intervention and implementation strategies will be delivered sequentially at 3 sites at 3-monthly intervals, after a prospective pre-intervention period of 8 months at the first site, with a total study duration and data collection period of 30 months.		
REDEEM-CAD Trial[34] (Ongoing) (NCT05366153)	The REDEEM-CAD study is a prospective cohort trial in Victoria and Tasmania assessing CAD screening in cancer survivors aged ≥ 40 years, ≥ 5 years post-treatment. Participants are stratified into low, intermediate, or high cardiovascular risk using clinical and biochemical assessment.		
	Intermediate risk: undergo coronary artery calcium (CAC) scoring; if CAC > 0 and < 400, CT coronary angiography (CTCA) is performed.		
	High risk or CAC > 400: offered preventive strategies (e.g., lipid-lowering therapy).		
	Low risk or CAC = 0: trial participation ends.		
	CTCA at baseline: repeat CTCA at 2 years.		
	Primary endpoint: prevalence of CAD by CAC in intermediate-risk survivors.		
	Secondary endpoint: change in coronary plaque volume at 24 months.		

Translationally, Australian investigators are advancing mechanistic and therapeutic research. The Heart Research Institute (HRI) is developing patient-derived pre-clinical cardio-oncology platforms for cardiotoxicity screening. The Newcastle Centre of Excellence in Cardio-Oncology identified Olaparib, a PARP inhibitor, as a dual-purpose drug conferring both anti-cancer efficacy and cardio-protection in-vitro and in-vivo, which has the potential to be a paradigm-shifting concept.

National registry infrastructure is also developing. The Australian Cardio-Oncology Registry (ACOR) prospectively captures adult and paediatric data across multiple sites for patients who develop CTRCD, with several ACOR sub-studies focusing on imaging and biomarkers are also in progress [3, 35, 36]. Australian investigators also contribute to and lead the Global Cardio-Oncology Registry (G-COR), which encompasses more than 120 sites across 20 countries and aims to collaborate worldwide data on cardio-oncology practices [37]. 

### Future Research

A co-ordinated research strategy has highlighted eight national priorities: improving understanding of cardiotoxicity mechanisms (for example through translational lab studies of novel cardio-protective therapies), developing less cardiotoxic cancer treatments and cardioprotective co-therapies, leveraging progressive technologies (from bioengineering solutions to artificial intelligence), progressing efforts to develop personalised patient care approaches and precision cardio-oncology whilst also ensuring research is both effectively designed and generalisable and finally research to enhance risk stratification and early detection [3]. 

There are several Australian cardio-oncology centres which are also contributing to large international collaborative research efforts. These include:

- COP-RCT will evaluate supervised withdrawal of cardioprotective therapy after recovery from CTRCD with advanced imaging to adjudicate relapse risk [38].
- SOCRATES is exploring if statins blunt ICI-associated atherosclerosis progression using serial coronary CT angiography [39].
- REDEEM-CAD systematises CAD screening in cancer survivors with CAC-guided CTCA and blinded core-lab plaque quantification [34].
- IMPACT-CVD (**ACTRN12624001005549)** is testing the implementation and efficacy of nurse led risk-guided cardio-oncology care across multiple centres.

Australia’s cardio-oncology research ecosystem – anchored by robust cancer registries, multi-disciplinary networks, and a strong clinical trials culture – positions it to lead global cardio-oncology innovation. Future priorities include equitable inclusion of Aboriginal and Torres Strait Islander cancer survivors and rural patients, improved national trial co-ordination, and suitable funding mechanisms [3]. Ultimately, translating these research priorities into evidence-based national policy will be critical to improving outcomes for all Australian cancer survivors.

## Section 6: Societal Involvement in Cardio-Oncology

Steady, coordinated work from professional societies and cross-disciplinary organisations have been a key contributor to the maturity of cardio-oncology in Australia. Over the last decade these groups have started to build a community of experts that continue to establish local standards, education, research networks, and advocacy within the field.

### The ICOS Australian Chapter

The Australian chapter of ICOS, established in 2019, provided the initial professional “home” for clinicians navigating the interface between cancer and cardiovascular disease. ICOS offered a shared language and conceptual model—risk stratification, surveillance pathways, and toxicity phenotypes—that helped to standardise heterogeneity in the way cardio-oncology care was delivered across centres. Its educational programs and weekly webinars supported upskilling opportunities. By connecting Australian centres into international working parties, it allowed Australian experts within the field to engage with international counterparts and ensured that protocols and consensus statements circulating globally could be adapted quickly to Australian referral pathways, funding rules, and survivorship services.

Importantly, by framing cardio-oncology as a distinct, collaborative craft rather than a cardiology consult service, the chapter improved clinician buy-in on both sides, smoothing the establishment of co-located clinics and shared care models. It also provided a “gold tier” centres of excellence system that recognised programs capable of comprehensive prevention, diagnostics, acute care of toxicity, and longitudinal survivorship follow-up. Those centres would later form an anchor network for national collaboration.

### The CSANZ–COSA Working Group

The formation of the CSANZ–COSA Working Group in 2020 marked the first formal collaboration between the peak cardiology society (Cardiac Society of Australia and New Zealand, CSANZ) and the peak oncology alliance (Clinical Oncology Society of Australia, COSA). This was a pivotal step in embedding cardio-oncology within the governance of both craft groups rather than relying on ad-hoc personal networks.

### The CSANZ Cardio-Oncology Committee

The establishment of the CSANZ Cardio-Oncology Committee in 2025 reflects a second maturation step: formal consolidation of the field within cardiology’s professional governance. While cardio-oncology is irreducibly multidisciplinary, locating a dedicated committee in CSANZ serves several pragmatic purposes:

### ACORES (est. 2024): A National Platform for Education, Research, and Advocacy

Launched in 2024, the Australian Cardio-Oncology Research, Education and Support network (ACORES) connects clinicians across cardiology, medical oncology, radiation oncology, and haematology with a mandate broader than any single society. Its remit spans:

- **Education.** ACORES will deliver the inaugural national flagship cardio-oncology conference in March 2026.
- **Research collaboration.** By linking a network of key cardio-oncology centres—including all Australian “gold tier” ICOS centres—ACORES lowers the barrier to multicentre observational cohorts, pragmatic trials, and implementation Science projects (e.g., e-pathway prompts for baseline risk stratification).
- **Advocacy and representation.** ACORES provides a unified voice to government, funders, and cancer agencies on issues such as equitable access to strain imaging and CMR, reimbursement for surveillance, and survivorship models integrating exercise physiology and cardiac rehabilitation.
- **Service networking.** The hub-and-spoke model helps smaller hospitals escalate complex cases, share protocols, and adopt tele-cardio-oncology consults without duplicating scarce expertise.

By design, ACORES complements—not replaces—society structures. Its cross-craft governance and operational focus make it an effective “engine room” for national projects, while formal societies supply standards, accreditation, and policy levers.

The development of cardio-oncology in Australia is a story of coordinated institution-building. The ICOS Australian Chap. (2019) supplied identity and international linkage; the CSANZ–COSA Working Group (2020) created the first durable bridge between craft groups; ACORES (2024) delivers a national, multidisciplinary network for education, research, and advocacy—linking all gold-tier ICOS centres; and the CSANZ Cardio-Oncology Committee (2025) embedded the field within cardiology’s quality and training ecosystem. Together, these organisations have transformed cardio-oncology from enthusiastic pockets of practice into a coherent national program with improving reach, consistency, and patient-centred outcomes.

## Section 7: Nursing, Pharmacy, and Allied Health Collaborations in Cardio-Oncology

Multidisciplinary care is central to modern oncology and cardiology practice, yet the integration of nurses, pharmacists, and allied health professionals within cardio-oncology teams remains underdeveloped [40–43]. 

Nursing forms the backbone of multidisciplinary teams (MDTs), providing clinical assessment, education, and continuity of care. Advanced practice and nurse practitioner–led models have demonstrated improved cardiovascular outcomes, reduced readmissions, and high patient satisfaction across chronic cardiac conditions such as heart failure and atrial fibrillation [44–48]. 

Within oncology, nurse practitioners have long been integral to care coordination, symptom management, and survivorship programs [49–53]. Their roles in cardio-oncology MDTs are now gaining recognition, offering opportunities to extend proven cardiovascular nursing models to cancer populations requiring long-term cardiovascular surveillance [50, 54, 55]. Early evidence indicates that nurse-led cardio-oncology services can deliver effective, cost-efficient care, reducing hospital utilisation and improving patient engagement [46, 56, 57]. 

Pharmacists are equally critical to cardio-oncology care, ensuring safe prescribing and minimising drug–drug and drug–disease interactions between cardiovascular and anticancer therapies [58–61]. Polypharmacy—common among cancer patients—increases the risk of adverse medication events [62, 63]. The American Heart Association recognises pharmacists as essential MDT members for managing pharmacologic interactions and optimising cardioprotective therapy [64]. Studies have shown that patients with cancer and cardiovascular comorbidities remain undertreated with key cardioprotective agents, underscoring the need for pharmacist involvement [65]. Clinical trials such as TITAN demonstrate how embedded pharmacists support medication review and cardiovascular risk management within multidisciplinary cardio-oncology programs [66–68]. 

Allied health professionals—including physiotherapists, dietitians, and psychologists—contribute to comprehensive survivorship care through exercise, nutrition, and psychosocial interventions. Cardio-oncology rehabilitation has emerged as a vital extension of cancer survivorship, improving cardiorespiratory fitness, quality of life, and long-term cardiovascular outcomes [69, 70]. 

Collectively, these roles highlight the need for structured, multidisciplinary cardio-oncology models that integrate nursing, pharmacy, and allied health expertise to deliver holistic, sustainable, and patient-centred care across the cancer continuum.

## Section 8. Barriers to Cardio-Oncology Service Access in Australia

Despite growing awareness and research, access to dedicated cardio-oncology services in Australia remains both limited and uneven. Several key factors such as geographic, population-specific, workforce-related, and systemic challenges pose the greatest barriers to delivering cardio-oncology care across Australia.

### Geography and Distance

One-third of Australians live in regional or remote areas outside major cities [71]. Specialized cardio-oncology clinics are presently concentrated in a few metropolitan centres (e.g. Sydney, Melbourne, Newcastle, Adelaide), creating access inequities associated with worse cardiovascular outcomes including significantly higher rates of cardiac events (such as admissions with acute coronary syndrome or arrhythmia) and higher all-cause and cardiovascular mortality [8, 72]. 

Telehealth and outreach services offer potential solutions [73]. The SMART-BREAST trial is an example of an innovative Australian initiative utilising smartphone based models of care to improve access and engagement for breast cancer patients in CVD risk reduction [7]. However, several telehealth issues such establishing stable internet access rurally, sourcing appropriate location settings for telehealth clinics and the need for in-person cardiac imaging limit their reach and impact [74]. Scalable outreach via regional satellite clinics, mobile services, and funded telehealth programs is needed to close the urban–rural gap.

### Population Health Disparities

Aboriginal and Torres Strait Islander Australians experience disproportionately high cardiovascular risk and poorer cancer outcomes, often due to later diagnosis and limited access to care [10, 75–78]. Culturally appropriate services and community driven services are scarce, and indigenous participation in trials remains extremely low. Similarly, socioeconomic and linguistic barriers restrict access for migrant and disadvantaged groups [77, 78]. Most patient education materials are English-only and not culturally tailored to Australia’s multicultural population. Proposed efforts to counter this include Indigenous-specific research modules and co-designed educational tools, but broader implementation is required [3]. 

### Workforce and Expertise

A shortage of trained cardio-oncology specialists limits service availability. Nurses and allied health professionals, who play a key role in cardio-oncology (for risk factor counselling, surveillance, rehabilitation), are also in short supply for this niche area and often lack specific training [27]. Many patients are managed by general cardiologists and oncologists without formal cardio-oncology training, leading to inconsistent approaches to screening, surveillance, and management of cardio-oncology conditions and delayed referrals to tertiary specialist cardio-oncology services. Tertiary clinics face long wait times and limited capacity, and primary-care practitioners have had to increasingly fill the gap despite minimal dedicated training [79]. 

Expanding the workforce through funded training pathways, dedicated coordinator roles, and nurse-led models is essential. The nurse-led cardio-oncology pilot in Newcastle, which received a national innovation award, demonstrates the promise of such approaches [42]. 

### Systematic Barriers and Constraints

Australia’s fragmented healthcare structure and funding model compound these barriers. Cardiology and oncology have typically operated in silos with no universal pathway for cardiac evaluation during cancer care. A lack of national cardio-oncology guidelines led to variable practices across institutions. A lack of clear referral criteria, reimbursement incentives, and integration between hospital data systems further impeded co-ordinated care.

Long-term survivorship follow-up in another gap, as many patients are lost between oncology discharge and primary-care review. General practitioners often do not have access to detailed treatment summaries outlining cardiovascular risks, resulting in under-prescription of cardioprotective therapy. Integrated survivorship plans and shared electronic communication between teams are urgently needed [79, 80]. 

### Addressing the Barriers

**Fig. 3:**
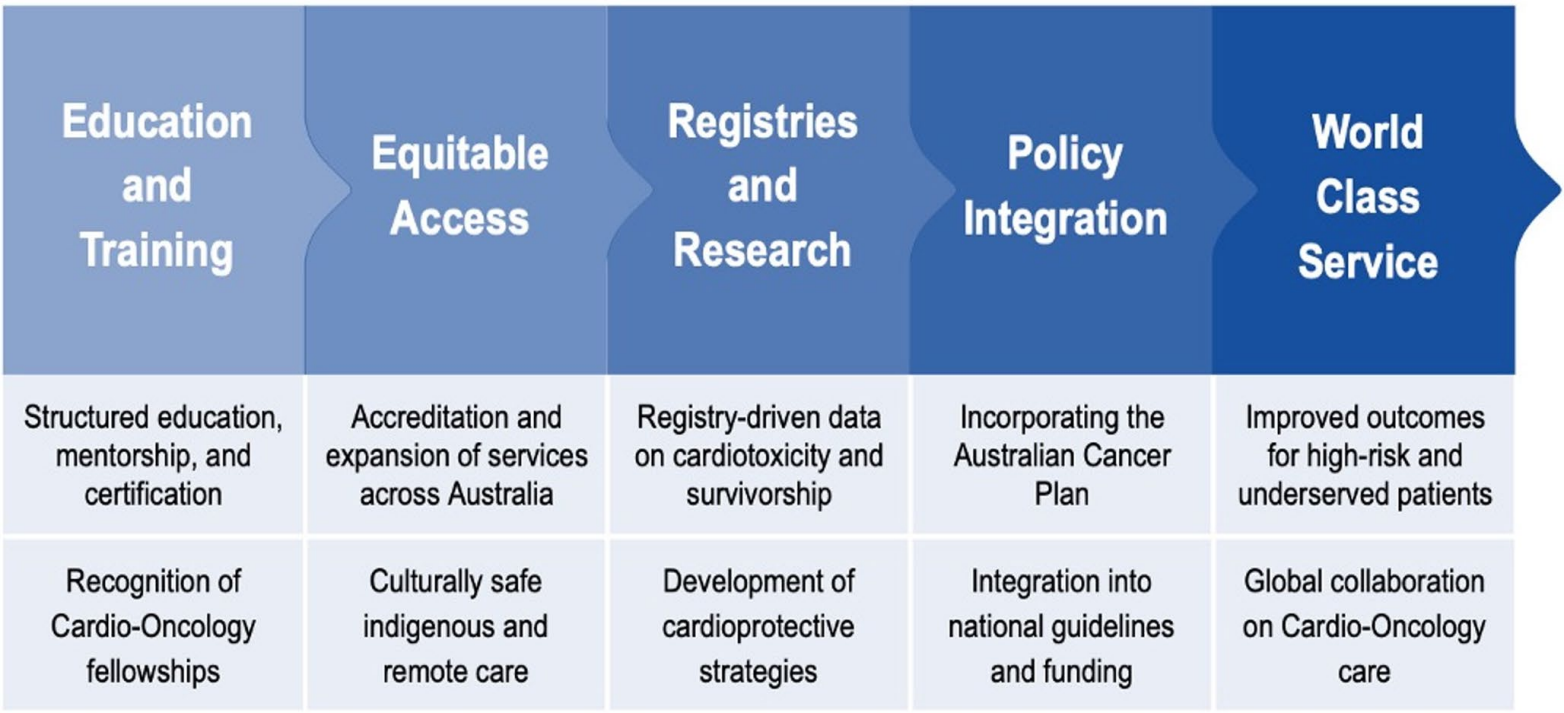
A proposed national roadmap for the next phase of Australian cardio-oncology

Access equities in cardio-oncology stem from geography, population vulnerability (of indigenous, low socioeconomic, and rural and remote cohorts), workforce limitations, and systemic fragmentation. Promising national initiatives, including the forthcoming COSA/CSANZ quality standards and the Australian Cancer Plan, aim to embed cardio-oncology into routine cancer care [81]. A proposed national roadmap for the next phase of Australian cardio-oncology is summarised in Fig. 3.

Key strategies include:

- Co-designed services with consumer and Indigenous health input.[3]
- Telehealth networks and regional outreach clinics.
- Workforce development
- Policy reform and sustainable funding for integrated programs.
- Enhanced data infrastructure via registries and big-data linkage.

## Section 9: Conclusion

Cardio-oncology in Australia has progressed from disparate grassroots initiatives to an increasingly recognised subspecialty, driven by rising cancer survivorship and the growing need to address cardiovascular toxicity from cancer therapies. Over the past decade, structured clinics have been established across several tertiary centres, delivering care that spans early risk stratification, prevention and management of overt cardiotoxicity, and long-term survivorship surveillance. National collaborations have strengthened the framework for clinical practice, research, and education, reflecting the maturation of the field.

Nonetheless, significant challenges remain. Access to dedicated services is concentrated in metropolitan areas, leaving rural and Indigenous communities underserved. Geographic inequities are compounded by systemic barriers such as limited workforce capacity, absence of sustainable funding streams, and lack of integrated clinical pathways between oncology and cardiology. Formal training pathways are still developing, with most clinicians acquiring expertise through informal routes rather than accredited programs. Without strategic expansion, service capacity will continue to lag behind demand as newer cancer therapies become standard of care. Furthermore, multidisciplinary integration of allied health, nursing, and pharmacy roles remains inconsistent.

Despite these limitations, Australian clinicians and researchers have influential contributions to global cardio-oncology. Landmark studies such as SUCCOUR, BREXIT, and SMART-BREAST, along with the establishment of the Australian Cardio-Oncology Registry (ACOR), and participation in international collaborations such as G-COR, underscore Australia’s leadership in evidence generation and implementation.

Future priorities include embedding cardio-oncology into national health policy, ensuring equitable access through outreach and telehealth, developing structured training and accreditation, and integrating culturally appropriate models of care for Indigenous Australians.

The trajectory of cardio-oncology in Australia illustrates both remarkable progress and clear opportunity. By addressing workforce, access, and system-level challenges, while leveraging its strong research and collaborative networks, Australia is well positioned to deliver world-class, equitable cardio-oncology care, ensuring that every cancer survivor receives optimal cardiovascular health support throughout cancer treatment and beyond.

## Data Availability

No datasets were generated or analysed during the current study.
